# Seroprevalence of bluetongue disease in sheep in west and northwest provinces of Iran

**Published:** 2013

**Authors:** Mohammad Khezri, Seyed Mahmud Azimi

**Affiliations:** 1 *Department of Veterinary Medicine, Agricultural and Natural Resources Research Center of Kurdistan, Sanandaj, Iran;*; 2 *Department of Foot-and-Mouth Disease Vaccine, Razi Vaccine and Serum Research Institute, Karaj, Iran.*

**Keywords:** Bluetongue, C-ELISA, Iran, Seroprevalence, Sheep

## Abstract

The objective of this study was to describe the seroprevalence rates of bluetongue virus (BTV) in sheep in west and northwest provinces of Iran. Bluetongue virus, an economically important orbivirus of the *Reoviridae* family, causes a hemorrhagic disease mainly in sheep and occasionally in cattle and some species of deer. Bluetongue virus is transmitted between its mammalian hosts by certain species of biting midges (*Culicoides spp*.) and it can infect all ruminant species. Overall, 26 serotypes have been reported around the world. Due to its economic impact, bluetongue (BT) is an Office of International des Epizooties (OIE)-listed disease. A total of 756 sera samples collected during 2007-2008, were available. Sera were tested with competitive enzyme-linked immunosorbent assay (C-ELISA). The seroprevalence rate in sheep was 40.87%. The rate of positivity in sheep in west and northwest was 46.10% and 33.75%, respectively. The highest prevalence of antibodies in serum was in West Azerbaijan (64.86%), and lower was in Ardabil (23.77%).

## Introduction

**Fig. 1:**
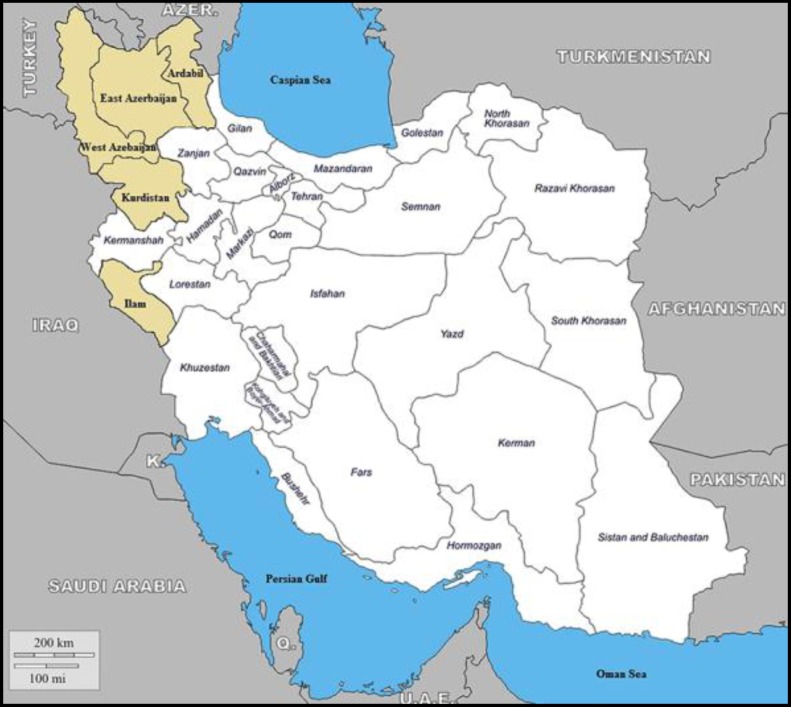
Map of Iran showing the location of areas where the present study was conducted.

Bluetongue (BT) is an insect-borne viral disease to which all species of ruminants are susceptible. It occurs mostly during periods of high temperature and rainfall, and usually disappears with the first frost or severe cold weather.^1^ Blue-tongue virus (BTV), the causative agent of BT of ruminants, have now been identified on all continents except for Antarctica.^2^^,^^3^ Bluetongue virus is transmitted between its ruminant hosts almost exclusively through the bites of the females of vector species of the *Culicoides spp.* biting midge.^4^^,^^5^ Specifically, BTV exists in an extensive band that includes tropical, subtropical, and temperate regions of the world between latitudes of approximately 40˚ north and 35˚ south. Exceptions are regions of Asia and western north America, where BTV infection of ruminants occurs as far as 50˚ north and, recently, northern Europe.^6^^,^^7^ However, the distribution of specific insect vectors and different BTV serotypes differ remarkably throughout the world, so specific vector exist with specific constellations of BTV serotypes and topotypes in relatively distinct global ecosystems.^2^^,^^8^^,^^9^ Although BTV is an orbivirus, it can occasionally be transmitted via seminal fluid and across the placenta.^10^ Diagnostic tests are a major component of the success in any surveillance system. Wide varieties of tests are capable of detecting BTV-specific anti-bodies. These include agar gel immune-diffusion (AGID), hemagglutination-inhibition (HI), complement fixation (CF) and enzyme-linked immunosorbent assay (ELISA) either blocking ELISA or competitive ELISA (C-ELISA) which are serogroup-specific and serum neutralization (SN) test which is serotype-specific.^11^ Only AGID and C-ELISA are recommended as prescribed tests for international trade in the Office of International des Epizooties (OIE) Manual of Standards for Diagnostic Tests and Vaccines.^12^ Reports on BT outbreaks in the second semester of 2008 in Iran,^13^ sera-positive herds in Turkey,^6^^,^^14^ BT infection in Saudi Arabia, and outbreaks in Oman, and the Palestinian Autonomous Territories are among the most recent incidents of the disease in the region.^15^ There have been few recent published studies from the region. Thus, we designed a study to evaluate the prevalence and distribution of serum antibodies to BTV in sheep in this area of Iran (Fig. 1).

## Materials and Methods

**Animals. **In this study, for detection of specific antibodies to BTV in sheep blood serum samples by C-ELISA, 756 sheep blood samples collected from likely seropositive area in the west and northwest of Iran has investigated between June 2007 and September 2008. 

**C-ELISA. **Anti-BTV antibodies were detected in serum samples by group specific, C-ELISA kit (ID-Vet, Monpellier, France). The test based on competitive between test sera and an anti-VP7 for a VP7 antigen previously bound to the solid phase of ELISA plate.

## Results

Seroprevalence of bluetongue in west and northwest was shown in Table 1. The results showed that the seropositive rate in sheep over the whole study area was 40.87%. Presence of anti-bluetongue antibodies was found highest in west. The highest prevalence of antibodies was in West Azerbaijan (64.86%), and lower (23.77%) was in Ardabil (Table 1).

**Table 1:** Seroprevalence of bluetongue antibodies in sheep from the studied regions of Iran.

	**Number ** **of serum samples**	**Number ** **of seropositive**	**Positive rate (%)**
***Studied regions***			
**Northwest**	320	108	33.75
**West**	436	201	46.10
***Total***	756	309	40.87
***Studied Provinces***			
**Ardabil**	122	29	23.77
**East Azerbaijan**	198	79	39.89
**West Azerbaijan **	74	48	64.86
**Kurdistan**	151	63	41.72
**Ilam**	211	90	42.65
***Total***	756	309	40.87

## Discussion

It is worth mentioning that the situation of diagnosis of this virus in neighboring countries and the Middle-East (except Turkey and Occupied Palestine) is not better than our country. In such country as Saudi Arabia, Syria, Yemen and Pakistan, the presence of virus has been documented only relying on serological tests.^6^^,^^7^^,^^16^ According to recent studies, there is an evidence of occurrence of BT disease in tropical and subtropical countries (such as Iran). In such areas generally, the disease appears sub clinically and does not attract attention. In such circumstances, the presence of the virus often confirms via serological evidence. It should be mentioned that in such foci, in spite of unrevealed disease and manifestation, sometimes sudden incidence of acute forms of the disease sustain a loss.^17^^-^^19^

In Iran, identification of BTV in suspected cattle and sheep based on clinical manifestations was performed. However, there are some limitations and problems. First, it should be considered that clinical expression of BTV regarding strain and virus intensity, cattle race and environmental condition varies from per acute to subclinical. Second, symptoms of disease in sheep can be mistaken with those of other viral much diseases and even some of the non-viral diseases.^19^ The objectives of the used C-ELISA test were both to confirm the BTV infected status of sheep in suspicious holdings in west and northwest of Iran (Diagnostic test). Thus, its use has been more and more often abandoned and replaced by C-ELISA tests, which are rapid and easier to use, more sensitive and specific.^20^ In this study, the prevalence rate of BT antibodies in sheep was 40.87%. In other countries, the prevalence was as follows: 21.40% in Kazakhstan,^6^ 29.59% in southeastern Turkey,^14^ 62.69% in India,^21^ 54.10% in Saudi Arabia,^22^ and 48.70% in Pakistan.^16^ Climatic factors play an important role in the occurrence of BTV infection in animals and influence the size of vector populations and periods of their seasonal activity.^23^ An analysis of climatic data was used to model the potential distribution of *Culicoides imicola* in Europe, predicting that might have spread from Spain, Greece and Italy to some areas along the Croatian coast as well as to the coastal areas of Albania, Serbia, Montenegro, Bosnia and Herzegovina.^24^^-^^27^ Although more than 1000 species of *Culicoides spp*. are known worldwide, relatively few of these species have been incriminated as vectors of BTV.^28^ *Culicoides* from western Turkey in relation to bluetongue disease of sheep and cattle was reported.^29^ Species of vector insects that transmit BTV differ amongst regions, and are especially poorly characterized in the portions of Asia that are devoid of *C. imicola*, the traditional African-Asian vector of BTV.^2^^,^^7^^,^^29^ Animals entering the western border of Iraq to Iran can be a cause of high titers of antibodies against the bluetongue virus in west of Iran. The prevalence of BT antibodies in sheep in the northwest of Iran was 33.75%. Although BTV infection of sheep is clearly widespread in northwest of Iran, the specific virus serotypes and vector insects that occur within the region remain uncharacterized, as they are in adjacent countries such as Kazakhstan.^6^ The highest prevalence of BTV in sheep was in West Azerbaijan (64.86%) and Ilam (42.65%).

The results show that BTV infection is present in live-stock animals in province. Some similar studies have been carried out in different area of country that mostly reported prevalence in the similar study. For example, 76.44% in East Azerbaijan,^30^ 34.70% in West Azerbaijan,^7^ 33.33% in Kerman,^31^ 45.90% in Kurdistan, ^32^ 53.37% in Isfahan.^33^

Furthermore, BTV infection in sheep apparently is largely subclinical.^7^ During the BTV epidemics in Europe in 2008, Williamson and colleagues^34^ considered clinical signs for diagnosis of the disease. The results showed low specificity of this method. These researchers believe that sometimes clinical signs of BTV in sheep are mistaken with those of such diseases as foot and mouth disease (FMD), ovine rinderpest, contagious ecthyma, and hemonchosis.^35^^,^^36^ Iran located in the southeast of Europe makes it an important potential source of BTV strains and serotypes that might incur into adjacent areas.^5^^,^^37^

In conclusion, seroprevalence of BTV has been never before reported in many area of Iran (Ardabil and Ilam). As per our knowledge, this is the first study was evaluated the prevalence of antibodies to BTV in sheep in some provinces of Iran. The results showed that a high incidence rate of BT antibodies has been detected in sheep in Iran that indicate serological evidence of exposure to infection was widely distributed in some provinces. There are no restrictions on the movement of animals from one region to another within the country. Thus, outbreaks may also occur due to transportation of animals. Consequently, a well-defined control strategy for preventing and controlling the BTV may be based not only on vaccination plans and vector eradication but also restriction on the movement of animals from one region to another within the country. As a vaccination for BT is not implemented in Iran, a seropositive result indicates BT infection in domestic population.^4^^,^^31^ Further researches on the isolation and identification of BT virus in sheep are encouraged.

## References

[1] Bluetongue detected for the first time in Northern Europe. http://www.oie.int/en.

[2] Tabachnick WJ (2004). Culicoides and the global epidemiology of bluetongue virus infection. Vet Ital.

[3] Maclachlan NJ (2011). Bluetongue: History, global epidemiology, and pathogenesis. Prev Vet Med.

[4] Mellor PS, Widmann EJ (2002). Bluetongue virus in the Mediterranean Basin. Vet J.

[5] Saegerman C, Berkvens D, Mellor PS (2008). Bluetongue epidemiology in European Union. Emerg Infect Dis.

[6] Lundervold M, Milner-Guilland EJ, O’Callaghan CJ (2003). First evidence of blue tongue virus in Kazakhstan. Vet Microbiol.

[7] Jafari-Shoorijeh S, Ramin AG, Maclachlan NJ (2010). High seroprevalence of bluetongue virus infection in sheep flocks in West Azerbaijan, Iran. Comp Immunol Microbiol Infect Dis.

[8] Maclachlan NJ, Osburn BI (2006). Impact of bluetongue virus infection on the international movement and trade of ruminants. Am Vet Med Assoc.

[9] Balasuriya UB, Nadler SA, Wilson WC (2008). The NS3 proteins of global strains of bluetongue virus evolve into regional topotypes through negative (purifying) selection. Vet Microbiol.

[10] Schwartz-Cornil I, Mertens PPC, Contreras V (2008). Bluetongue virus: virology, pathogenesis and immunity. Vet Res.

[11] Afshar A (1994). Bluetongue: Laboratory diagnosis. Comp Immunol Microbiol Infect Dis.

[12] Bluetongue, manual of diagnostic tests and vaccines for terrestrial animals (mammals, birds and bees). http://www.oie.int/doc/ged/D7709.

[13] Principles of validation of diagnostic assays for infectious diseases, manual of diagnostic tests, and vaccines for terrestrial animals (mammals, birds and bees). http://www.baphiq.gov.tw/public/Attachment/922%2051%207212071.pdf.

[14] Gür S (2008). A serologic investigation of bluetongue virus in cattle, sheep and Gazella subgutturosa in south-eastern Turkey. Trop Anim Health Prod.

[15] (Final report of the 10th conference of the OIE regional commission for the middle). http://www.oie.int/eng/session2011/A_%20FR_2010PUB.pdf.

[16] Akhtar S, Djallem N, Shad G (1997). Bluetongue virus seropositivity in sheep flocks in North West Frontier province, Pakistan. Prev Vet Med.

[17] Basak AK, Grimes J, Gouet P (1997). Structures of orbi-virus VP7: implications for the role of this protein in the viral life cycle. Structure.

[18] Nikolakaki SV, Nomikou K, Koumbati M (2005). Molecular analysis of the NS3/NS3A gene of bluetongue virus isolates from the 1979 and 1998-2001 epizootics in Greece and their segregation into two distinct groups. Virus Res.

[19] Momtaz H, Nejat S, Souod N (2011). Comparisons of competitive enzyme-linked immunosorbent assay and one step RT-PCR tests for the detection of bluetongue virus in south west of Iran. African J Biotechnol.

[20] Reddington JJ, Reddington GM, MacLachlan NJ (1991). A competitive ELISA for detection of antibodies to the group antigen of bluetongue virus. J Vet Diagn Invest.

[21] Sreenivasulu D, Subba Rao MV, Reddy YN (2004). Overview of bluetongue disease, viruses, vectors, surveillance and unique features: the Indian subcontinent and adjacent regions. Vet Ital.

[22] Yousef MR, Al-Eesa AA, Al-Blowi MA (2012). High sero-prevalence of bluetongue virus antibodies in Sheep, Goats, Cattle and Camel in different districts of Saudi Arabia. Vet World.

[23] Ward MP, Thurmond MC (1995). Climatic factors associated with risk of seroconversion of cattle to bluetongue viruses in Queensland. Prev Vet Med.

[24] Gloster J, Mellor PS, Manning AJ (2007). Assessing the risk of windborne spread of bluetongue in the 2006 outbreak of disease in northern Europe. Vet Rec.

[25] Gubbins S, Carpenter S, Baylis M (2007). Assessing the risk of bluetongue to UK livestock: Uncertainty and sensitivity analysis of a temperature-dependent model for the basic reproductive number. J R Soc Interface.

[26] Wilson AJ, Carpenter S, Gloster J (2007). Re-emergence of bluetongue in northern Europe in 2007. Vet Rec.

[27] Withitann EJ, Mellor PS, Baylis M (2001). Using climate data to map the potential distribution of Culicoides imicola (Diptera: Ceratop-ogonidae) in Europe. Rev Sci Technol Off Int Epiz.

[28] Meiswinkel R, Gomulski LM, Delecolle JC (2004). The taxonomy of Culicoides vector complexes unfinished business. Vet Ital.

[29] Maclachlan NJ (2010). Global implications of the recent emergence of bluetongue virus in Europe. Vet Clin North Am Food Anim Pract.

[30] Hasanpour A, Mosakhani F, Mirzaii H (2008). Sero-prevalence of bluetongue virus infection in Sheep in East-Azerbaijan province in Iran. Res J Biol Sci.

[31] Mozaffari AA, Khalili M, Mashayekhi M (2012). The first survey for antibody against bluetongue virus in sheep flocks in Southeast of Iran. APJTB.

[32] Khezri M (2012). Seroprevalence of bluetongue virus anti-bodies in sheep in Kurdistan province in west of Ian. IJAVMS.

[33] Noman V, Kargar Moakhhar R, Shah Moradi AH (2006). A seroepidemiological survey for bluetongue virus anti-body in sheep and goats of Isfahan province, Iran, Isfahan, Iran. Isfahan agricultural and natural resources research center.

[34] Williamson S, Woodger N, Darpel K (2008). Differential diagnosis of bluetongue in cattle and sheep. In Practice.

[35] Elbers AR, Backx A, Ekker HM (2008). Performance of clinical signs to detect bluetongue virus serotype 8 out-breaks in cattle and sheep during the 2006-epidemic in The Netherlands. Vet Microbiol.

[36] Tan BH, Nason E, Staeuber N (2001). RGD tripeptide of bluetongue virus VP7 protein is responsible for core attachment to Culicoides cells. J Virol.

[37] Purse BV, Mellor PS, Rogers DJ (2005). Climate change and the recent emergence of bluetongue in Europe. Nat Rev Microbiol.

